# Unveiling antimicrobial resistance in Chilean fertilized soils: a One Health perspective on environmental AMR surveillance

**DOI:** 10.3389/fmicb.2023.1239761

**Published:** 2023-12-01

**Authors:** Marcela Fresno, Leonardo Pavez, Yanina Poblete, Alexandra Cortez, Talía Del Pozo

**Affiliations:** ^1^Núcleo de Investigaciones Aplicadas en Ciencias Veterinarias y Agronómicas, Facultad de Medicina Veterinaria y Agronomía, Universidad de Las Américas, Providencia, Santiago, Chile; ^2^Red CYTED-USCC. CYTED 412RT0117: Una Salud en Iberoamérica y El Caribe frente al cambio climático y la pérdida de biodiversidad, Santiago, Chile; ^3^Núcleo de Investigación en Ciencias Biológicas (NICB), Facultad de Medicina Veterinaria y Agronomía, Universidad de Las Américas, Providencia, Santiago, Chile; ^4^Departamento de Ciencias Humanas, Universidad Bernardo O’Higgins, Santiago, Chile; ^5^Facultad de Medicina Veterinaria y Agronomía, Universidad de Las Américas, Santiago, Chile

**Keywords:** One Health, AMR, ARG, fertilized soils, manure, environmental surveillance, Chile

## Abstract

Antimicrobial resistance (AMR) poses a significant threat to humans and animals as well as the environment. Within agricultural settings, the utilization of antimicrobial agents in animal husbandry can lead to the emergence of antimicrobial resistance. In Chile, the widespread use of animal-derived organic amendments, including manure and compost, requires an examination of the potential emergence of AMR resulting from their application. The aim of this research was to identify and compare AMR genes found in fertilized soils and manure in Los Andes city, Chile. Soil samples were collected from an agricultural field, comprising unamended soils, amended soils, and manure used for crop fertilization. The selected genes (*n* = 28) included genes associated with resistance to beta-lactams, tetracyclines, sulfonamides, polymyxins, macrolides, quinolones, aminoglycosides, as well as mobile genetic elements and multidrug resistance genes. Twenty genes were successfully identified in the samples. Tetracycline resistance genes displayed the highest prevalence, followed by MGE and sulfonamides, while quinolone resistance genes were comparatively less abundant. Notably, blaOXA, sulA, tetO, tetW, tetM, aac (6) ib., and intI1, exhibited higher frequencies in unamended soils, indicating their potential persistence within the soil microbiome and contribution to the perpetuation of AMR over time. Given the complex nature of AMR, it is crucial to adopt an integrated surveillance framework that embraces the One Health approach, involving multiple sectors, to effectively address this challenge. This study represents the first investigation of antimicrobial resistance genes in agricultural soils in Chile, shedding light on the presence and dynamics of AMR in this context.

## Introduction

Antimicrobial resistance (AMR) is a growing public health concern worldwide, affecting both human and animal health, as well as the environment (Boden and Mellor, 2020; Shawver et al., 2021). The potential causes of global AMR comprise excessive use of antibiotics in animals, misuse of antibiotics in humans, over-the-counter antibiotic availability, the growth of international travel, poor sanitation and hygiene, and the discharge of unmetabolized antibiotics or their residues into the environment via manure, urine, and feces (Aslam et al., 2018). AMR occurs when bacteria, viruses, fungi, and parasites develop resistance to the drugs that are commonly used for their treatment (Puvača, 2022). As a consequence, AMR limits the effectiveness of antibiotics, leading to longer hospital stays, increased morbidity and mortality, and increased healthcare costs, and have a detrimental impact on the Gross Domestic Product of countries (Boeckel et al., 2019; Abushaheen et al., 2020). As a result, infections related to AMR are becoming a renewed threat to public health (Ventola, 2015). It is crucial to address this issue by implementing effective strategies to prevent the spread of antimicrobial resistance and promote the responsible use of antibiotics to ensure the continued efficacy of these life-saving drugs (Roca et al., 2015).

AMR can manifest as antimicrobial-resistant bacteria and antimicrobial resistance genes (ARGs), both of which have the potential to enter and persist in ecosystems through various pathways. These pathways include soil, water, crops, and gut microbial communities of wildlife, livestock, and humans (Du and Liu, 2012; He T. et al., 2021). In agricultural settings, the use of antimicrobial agents in animal husbandry can lead to the selection and proliferation of resistant bacteria and ARGs (Thanner et al., 2016; Mshana et al., 2021). These resistant organisms can subsequently contaminate the environment through animal waste, runoff, and irrigation (Roe and Pillai, 2003; Chemaly et al., 2014; Adegoke et al., 2016). Additionally, animals serve as reservoirs and vectors of AMR genes, facilitating its transmission and persistence between domestic and wild animals, and environments (Graham et al., 2019; Bennani et al., 2020). Humans play a crucial role in this dynamic relationship, by actively participating in activities such as farming, food production, and recreational pursuits, and wastewater management (Fouz et al., 2020; Jadeja and Worrich, 2022). These activities not only involve interactions with animals and their environments, but also have the potential to contribute to the dissemination of AMR genes in the environment and among animal populations (Thakur and Panda, 2017).

Animal-derived organic amendments, such as manure and compost, are commonly used in Chilean agriculture (Infante and San Martín, 2016). The use of integrated nutrient management practices, which include the use of organic manures, has been found to improve soil physical, chemical, and biological properties, resulting in enhanced crop productivity and better quality of crop produce (Rani et al., 2015; Shakoor et al., 2021). However, the use of these amendments can also contribute to the development of AMR in soil bacteria, which can have negative impacts on human and animal health (Zhang et al., 2020; Yang et al., 2021). Recent research has focused on the effects of different types of organic amendments on soil health and greenhouse gas emissions (Kalus et al., 2019; Urra et al., 2019). Nonetheless, limited information is available on the emergence of AMR resulting from the application of animal-derived organic amendments in fertilized soils in Chile.

The detection of antimicrobial residues in the environment is frequently linked to the usage of commonly employed antimicrobials in animal production and human health, that through various pathways, including inadequate disposal of antimicrobials into sewage systems or solid waste management, discharge of treated or untreated wastewater intro water bodies, runoff from agricultural fields where manure is applied, and leaching from livestock waste storage facilities, can enter the environment (Bian et al., 2015; Monteiro et al., 2016; Lima et al., 2020). Antibiotics, including tetracyclines, macrolides, fluoroquinolones, and sulfonamides, are among the most prevalent antimicrobial residues identified (Huong et al., 2020; Yang et al., 2021).

The use of antimicrobial agents in animal husbandry and agriculture should be carefully managed to minimize the risk of AMR development and spread. Understanding the prevalence and distribution of resistance genes in livestock manure and fertilized soils can help guide the development of strategies to minimize the spread of antibiotic resistance. This research article aims to identify and compare antimicrobial resistance genes detected in unamended soils, amended soils, and manure the Los Andes city, Chile. By investigating the relationship between animal amendments and AMR in Chilean fertilized soils, this study intends to shed light on the potential impact of this practice on the health of humans, animals, and the environment, and highlight the importance of a One Health approach to tackle this issue.

## Methods and results

### Samples

The soil samples were collected from an agricultural field located in the city of Los Andes, Valparaiso region, Chile, where peach crops are cultivated. The different soil samples consisted in: a) unamended soils, which have not been fertilized in September 2021 (32°50′40.1”S 70°33′36.9”W); b) soils amended, in September 2021, with organic fertilizers of animal origin (32°53′31.7”S 70°35′34.8”W); c) organic amendments of animal origin used for crop fertilization. The organic amendments of animal origin corresponded to samples of cow and horse manure, acquired in August–September 2021 locally by the producer from the agricultural field, and were not traceable. For each condition, four samples with five technical replicates were considered.

The soil samples were obtained from five different locations at a depth of 0–20 centimeters using the envelope method (Buta et al., 2021). The samples were collected using a sterile metal spatula and transferred to transparent polyethylene bags labeled as NascoTM Whirl-PakTM. All samples were transported under refrigeration conditions (4°C) to the Research Laboratory of Universidad de Las Americas Campus Providencia for processing, where they were stored at a freezing temperature (−20°C) until analysis.

### DNA extraction from soils and organic amendments

The collected samples were air-dried overnight at room temperature (20–25°C) and sieved to remove particles larger than 2 mm prior to their utilization (Barrios et al., 2021). Genomic DNA (gDNA) extraction was performed in triplicate for each sample. To obtain gDNA from soil and animal-derived organic amendments, the DNeasy^®^ PowerSoil^®^ Pro Kit (Qiagen, Germany) was used following the manufacturer’s instructions. All gDNA samples were stored at −20°C until further analysis. DNA concentration of each sample was measured using a SPECTROstar^®^ Nano absorbance plate reader (BMG Labtech), according to the manufacturer’s instructions. The positive controls employed consist of ARG adquired from various bacterial sources, encompassing both pathogenic and environmental bacteria, that had been previously isolated (Fresno et al., 2013; Retamal et al., 2015).

### Identification of antimicrobial resistance genes

Genomic DNA (0.5 ng/μL) underwent quantitative real-time PCR (qPCR) analysis to determine genes associated with resistance to different selected (*n* = 28) antimicrobials and horizontal gene transfer. Genes associated with resistance to beta-lactams (*blaCTX-M-04, blaTEM, blaOXA, blaSHV*), tetracyclines (*tetA, tetB/P, tetC, tetG, tetM, tetO, tetW, tetX*), sulfonamides (*sul1, sul2, sulA*), polimyxins (*mcr-1*), macrolides (*ermB, ermC, ermQ*), quinolones (*gyrA, aac (6′)-ib, qepA*), aminoglycosides (*aadA9*), mobile genetic elements (MGE) (*intI1, intI2, dfrA1*) and multidrug resistance (*mexF, oprJ*) were determined. The details of the primers and references of the used genes can be found in Supplementary Table S1.

A reaction mixture (total volume: 25 μL) was used, consisting of 12.5 μL of Brilliant II SYBR GREEN qPCR Master Mix (Stratagene), 0.5 μL of each specific forward and reverse primer for each gene, 11 μL of high-purity sterile water (Roche Diagnostics), and 0.5 μL of extracted DNA. All genes selected for the study were analyzed in triplicate. Reactions were performed using an AriaMX real-time PCR instrument (Agilent Technologies^®^). The thermal cycling conditions used consisted of one cycle at 95°C for 10 min, followed by 40 cycles of 30 s at 95°C and 1 min at 60°C. The Pfaffl method (Pfaffl, 2001) was used to determine the expression of ratio between investigated samples. Analysis were based on the values of the threshold cycle (Ct), calculated according to the efficiency of the reaction for each pair of primers, estimated using LinReg software (Ruijter et al., 2009). The data obtained were normalized with the relative abundance of 16S rRNA gene to present the frequency of genes in each of the investigated samples (Suzuki et al., 2000).

### Statistical analysis

Statistical analyzes were conducted in R-Studio software Version 1.3.1093. The results of ARG differences were analyzed according to their sample of origin. In addition, an antimicrobial resistance gene profile was determined based on their presence or absence. Statistically significant differences between samples were determined using the non-parametric Kruskal-Wallis test, results were considered statistically significant at a significance level of *p* < 0.05. A Principal Component Analysis (PCA) was conducted to explore the relationships within the dataset, the Pearson correlation coefficient was employed to assess the relationship between the variables.

## Results

**Figure 1:**
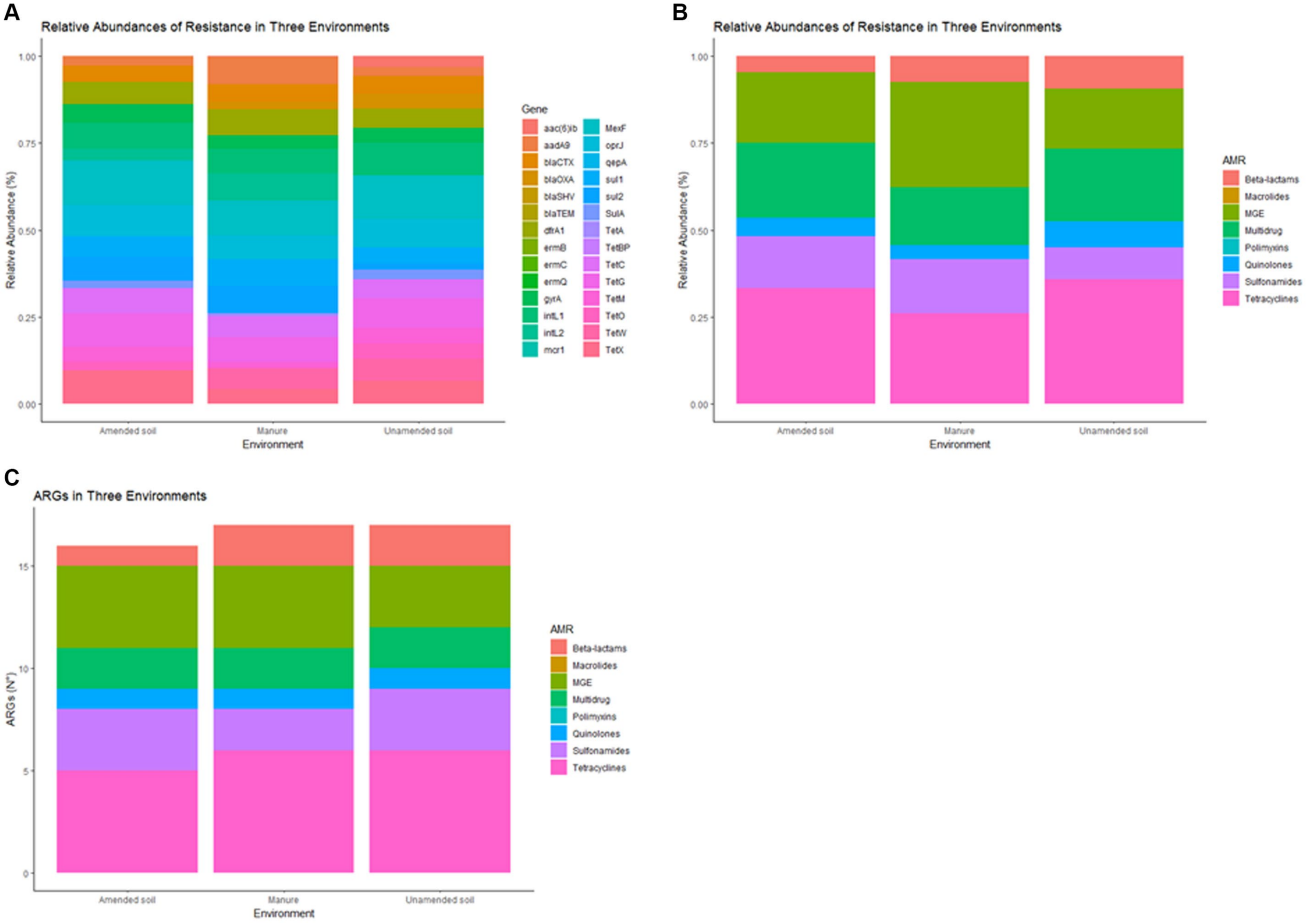
Relative abundance of antimicrobial resistance genes (ARGs) in non-amended soils, amended soils and animal-derived organic amendments, by gene **(A)** and by antimicrobial group **(B)**, antimicrobial resistance genes (N°) in non-amended soils, amended soils and animal-derived organic amendments **(C)**.

In this study, out of the 28 ARGs, 20 were identified and detected (Supplementary Table S1), in both amended and unamended soils, and animal manure. Tetracycline resistance genes were predominantly observed in both types of soils, followed by mobile genetic elements and multidrug resistance genes (Figures 1A,B). Conversely, when analyzing animal manure, the primary genes identified were MGE, followed by tetracycline and multidrug resistance genes (Figures 1A,B).

Significant statistical differences (p < 0.05) were observed when comparing the relative abundance of different groups of antimicrobial resistance genes (ARGs) between each condition (Figure 1B). Specifically, mobile genetic element (MGE) genes and genes associated with multidrug resistance exhibited notable differences. MGE genes (*dfrA1, aadA9, intL2*) were predominantly identified in manure samples, while genes related to multidrug resistance (*MexF, oprJ*) were more prevalent in the unamended soil samples. The most correlated ARG abundance profiles were amended and unamended soil samples, according to Pearson’s correlation (*r* = 0.9486).

The analysis of samples collected from animal manure and unamended soils revealed a significantly higher abundance of ARG compared to samples from amended soils, as illustrated in Figure 1C. Among all tested conditions, tetracycline resistance genes exhibited the highest number of detections, followed by mobile genetic elements and sulfonamides. In contrast, the presence of genes related to quinolone resistance was found to be relatively scarce in the samples.

**Figure 2:**
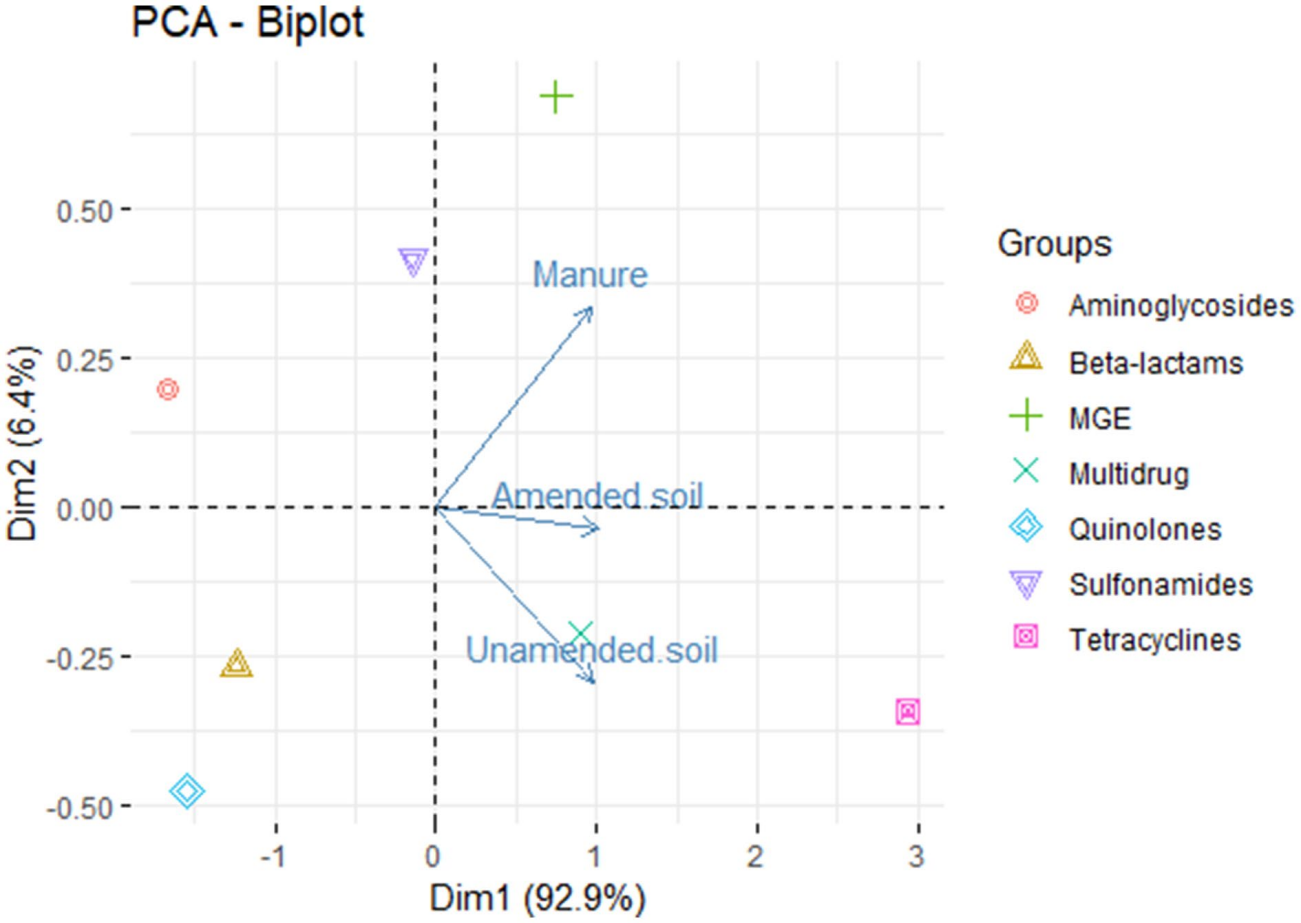
Principal component analysis of relative abundances of antimicrobial resistance groups in non-amended soils, amended soils and animal-derived organic amendments.

The principal component analysis shows that the first component explains 92.8% of the variance (Figure 2). This indicates that the ARGs observed in the samples do exhibit variation based on their origin, i.e., amended soils, unamended soils, and animal manure. We detected those genes conferring resistance to aminoglycosides, sulfonamides and MGE are mainly found in animal manure samples, while genes conferring resistance to tetracyclines and multidrug related genes are associated with both amended and unamended soils.

None of the samples showed the presence of genes associated with polymyxin resistance (*mcr-1*) or macrolide resistance (*ermB, ermC, ermQ*). Additionally, the genes *tetB/P, blaTEM, blaSHV,* and *qepA* were not detected in any of the samples (Supplementary Table S2).

## Discussion and conclusion

Antimicrobial resistance genes have the potential to be transmitted from animals and humans to the environment, where they can persist and spread (Iwu et al., 2020; Buta et al., 2021). The soil microbiome has the capacity to serve as a reservoir for these resistance genes, facilitating the perpetuation of antimicrobial resistance within the surrounding environments (Pu et al., 2019).

The use of fertilizers in agriculture offers various advantages, such as waste recycling, replace chemical fertilizers, enhanced soil quality, and reduced production expenses, among others (Ganesan, 2022). However, the use of animal amendments as fertilizers can introduce pathogens from animal feces into the environment (Zhang et al., 2020). Additionally, manure can contain antimicrobial residues, bacteria (including commensals) carrying resistance genes, and the resistance genes themselves, which can eventually contaminate the environment, water bodies, and soil (den Meersche et al., 2019; Lima et al., 2020). Moreover, crops cultivated in these amended soils have the potential to acquire these resistance genes, thereby increasing the likelihood of antimicrobial resistance transmission to humans and animals through the consumption of these crops (He L.-Y. et al., 2021; Huang et al., 2021).

In livestock production, the most frequently found ARGs are related to sulfonamide resistance (*sul*) (Makowska et al., 2016), this finding is consistent with the results obtained in this study, which identified the presence of all genes associated with sulfonamide resistance. It is important to understand that the detection of ARG in agricultural soils extends beyond regional borders, given the global nature of AMR and, specifically, *sul* genes can originate from different sources (Heuer et al., 2011; Chaturvedi et al., 2021). Tetracycline resistance genes (*tet*), detected in this study, are widely distributed in different pathogenic and environmental bacteria and are often detected in wastewater treatment plants, soils, surface waters, and groundwater (Chee-Sanford et al., 2001; Li et al., 2010; Wang et al., 2017). In agreement with this study, different authors have demonstrated the presence and persistence of multidrug-related genes and mobile genetic element genes in soil samples (Fricke et al., 2008; Zhang et al., 2020; Delgado-Baquerizo et al., 2022). The extensive use of antimicrobials in agriculture, including the application of animal manure and organic amendments, has been identified as a major contributing factor to the dissemination and maintenance of multidrug resistance genes in the environment (Iwu et al., 2020). The high prevalence of multidrug-related genes and mobile genetic elements in agricultural soils highlights the critical role of the environment as a reservoir and potential source of resistance genes (He et al., 2020; Lima et al., 2020), emphasizing the need for effective strategies to mitigate the environmental dissemination of antimicrobial resistance. The search for MGE provides insights for AMR prevention and control strategies that limits the acquisition and spread of AMR (Gillings, 2014; Hendriksen et al., 2019; Vrancianu et al., 2020). The identification of ARG in Latin America constitutes poses a substantial risk to food safety and security in this region (Reichert et al., 2019). While there is limited data on the occurrence of ARG in South America, studies have shown that clinically relevant ARG are more abundant in low- and middle-income settings in Africa, Asia, and South America, compared to high-income countries (Fouz et al., 2020).

In this study, the presence of antimicrobial resistance genes (ARGs) was compared among amended soils, unamended soils, and animal manure. Unamended soils referred to soils that had not received fertilization in the current year but had been previously amended in other seasons. These unamended soils should not be considered as completely clean since they carry a significant ARG load due to previous amendments. Interestingly, certain genes (such as *blaOXA, sulA, tetO, tetW, tetM aac (6) ib, intL1*) exhibited higher frequencies in unamended soils (Figure 1C). This finding suggests the potential persistence of these genes within the soil microbiome, contributing to the perpetuation of antimicrobial resistance in those environments over time (He L.-Y. et al., 2021). Conversely, genes including *blaCTX, sul1, sul2, tetA, dfrA1, aadA9* and *intL2* were detected at higher frequencies in animal manure (Supplementary Table S2). This observation is consistent with the expectation that manure would contain a greater abundance of ARGs, considering the association of farm animals, such as cows and horses from which the manure originated, with antimicrobials in animal production systems (Zhu et al., 2013; Xie et al., 2018). The soil microbiome can harbor ARG from various sources over time or even be the origin of these ARG (Colomer-Lluch et al., 2011; Forsberg et al., 2012). This implies that, regardless of whether soils receive fertilization or not, that may possess the potential to disseminate ARG to animals, plants, and humans (Forsberg et al., 2014; Cycoń et al., 2019). To prevent and mitigate this risk, an integrated approach is essential, which encompasses the management of animal-derived fertilizers, as well as addressing contamination sources like irrigation water, wastewater, and different animals, both wild and domestic.

There are various abiotic factors present in the soil, including pH, moisture content, heavy metals, temperature, and others, that play a significant role in determining the abundance of antibiotic resistance genes (Liu W. et al., 2021). These factors exert their influence by affecting the succession of bacterial communities in the soil microbiome, as well as the presence of mobile genetic elements (Liu B. et al., 2021). This could potentially elucidate the mechanisms responsible for the preservation of genes within unamended soils across successive seasons. Interestingly, resistance genes, when grouped by resistance type, were found to vary among the environments analyzed (Figure 2), where a relationship is seen between unamended and amended soils and multidrug resistance related genes and tetracyclines resistance genes, and between sulfonamides, aminoglycosides and MGE with animal manure. However, it is important to note that further analysis and examination of additional factors that may provide a more comprehensive understanding of the factors influencing resistance variations in the samples.

In the context of this study, it is important to note that the potential for contamination from human and animal sources cannot be ruled out, given the possible influence of various environmental reservoirs, such as wastewater and run-off from livestock facilities and agriculture (Holvoet et al., 2013; Berglund, 2015). The surveillance of antimicrobial resistance needs to consider the various stakeholders involved in the spread and persistence of AMR (Roca et al., 2015). The One Health approach acknowledges the interdependence of animals, humans, foods, and the environment, in the transmission and amplification of AMR. Therefore, it is crucial to adopt an integrated surveillance framework that embraces the One Health approach, involving multiple sectors, to effectively address the complex challenge of AMR (White and Hughes, 2019).

This study represents the first investigation of antimicrobial resistance genes (ARGs) in agricultural soils in Chile, and it stands as one of the early studies of its kind in South America. However, to derive more comprehensive and significant conclusions, further research involving a larger sample size is warranted. Additionally, future studies should consider the detection of antimicrobial residues across different environments, animals, and human sources, thereby expanding our understanding of this complex issue. By understanding and addressing the interplay between animals, humans, and the environment, we can effectively mitigate the persistence and expansion of AMR and safeguard public health and ecosystem integrity.

## Data availability statement

The raw data supporting the conclusions of this article will be made available by the authors, without undue reservation.

## Author contributions

MF: conceptualization and design of the study. MF, AC, and LP performed the experiment. MF and LP: collection of samples. AC and TP: sample preparation. MF: formal analysis and manuscript writing. MF and YP: manuscript editing. LP and YP: statistical and editing the manuscript. All authors contributed to the article and approved the submitted version.

## References

[ref1] Abushaheen M. A. Muzaheed A. J. Fatani M. A. Mansy W. George M. Acharya S.. (2020). Antimicrobial resistance, mechanisms and its clinical significance. Dis. Mon. 66:100971. doi: 10.1016/j.disamonth.2020.100971 32201008

[ref2] Adegoke A. A. Awolusi O. O. Stenström T. A. Adegoke A. A. Awolusi O. O. Stenström T. A. (2016). “Organic fertilizers: public health intricacies” in Organic fertilizers - from basic concepts to applied outcomes. eds. Larramendy M. L. Soloneski S. (London: IntechOpen)

[ref9001] Aslam B. Wang W. Arshad M. I. Khurshid M. Muzammil S. Rasool M. H.. (2018). “Antibiotic resistance: a rundown of a global crisis”. Infection and drug resistance, 11, 1645–1658. doi: 10.2147/IDR.S173867 30349322 PMC6188119

[ref9002] Barrios R. E. Bartelt-Hunt S. L. Li Y. Li X. (2021). “Modeling the vertical transport of antibiotic resistance genes in agricultural soils following manure application”. Environmental Pollution, 285:117480. doi: 10.1016/j.envpol.2021.117480 34087637

[ref3] Bennani H. Mateus A. Mays N. Eastmure E. Stärk K. D. C. Häsler B. (2020). Overview of evidence of antimicrobial use and antimicrobial resistance in the food chain. Antibiotics 9:49. doi: 10.3390/antibiotics9020049, PMID: 32013023 PMC7168130

[ref4] Berglund B. (2015). Environmental dissemination of antibiotic resistance genes and correlation to anthropogenic contamination with antibiotics. Infect. Ecol. Epidemiology 5:28564. doi: 10.3402/iee.v5.28564, PMID: 26356096 PMC4565060

[ref5] Bian K. Liu Y. H. Wang Z. N. Zhou T. Song X. Q. Zhang F. Y.. (2015). Determination of multi-class antimicrobial residues in soil by liquid chromatography-tandem mass spectrometry. RSC Adv. 5, 27584–27593. doi: 10.1039/C4RA13919D

[ref6] Boden L. Mellor D. (2020). “Epidemiology and ethics of antimicrobial resistance in animals” in Ethics and drug resistance: Collective responsibility for global public health. eds. Jamrozik E. Selgelid M. (Cham: Springer International Publishing), 109–121.

[ref7] Boeckel V. Thomas P. Pires J. Silvester R. Zhao C. Song J.. (2019). Global trends in antimicrobial resistance in animals in low- and middle-income countries. Science 365:eaaw1944. doi: 10.1126/science.aaw1944, PMID: 31604207

[ref8] Buta M. Korzeniewska E. Harnisz M. Hubeny J. Zieliński W. Rolbiecki D.. (2021). Microbial and chemical pollutants on the manure-crops pathway in the perspective of ‘one health’ holistic approach. Sci. Total Environ. 785:147411. doi: 10.1016/j.scitotenv.2021.147411, PMID: 33957582

[ref9] Chaturvedi P. Singh A. Chowdhary P. Pandey A. Gupta P. (2021). Occurrence of emerging sulfonamide resistance (Sul1 and Sul2) associated with Mobile Integrons-integrase (intI1 and intI2) in riverine systems. Sci. Total Environ. 751:142217. doi: 10.1016/j.scitotenv.2020.142217, PMID: 33181985

[ref10] Chee-Sanford J. C. Aminov R. I. Krapac I. J. Garrigues-Jeanjean N. Mackie R. I. (2001). Occurrence and diversity of tetracycline resistance genes in lagoons and groundwater underlying two swine production facilities. Appl. Environ. Microbiol. 67, 1494–1502. doi: 10.1128/AEM.67.4.1494-1502.2001, PMID: 11282596 PMC92760

[ref11] Chemaly R. F. Simmons S. Dale C. Ghantoji S. S. Rodriguez M. Gubb J.. (2014). The role of the healthcare environment in the spread of multidrug-resistant organisms: update on current best practices for containment. Ther. Adv. Infect. Dis. 2, 79–90. doi: 10.1177/2049936114543287, PMID: 25469234 PMC4250270

[ref12] Colomer-Lluch M. Jofre J. Muniesa M. (2011). Antibiotic resistance genes in the bacteriophage DNA fraction of environmental samples. PLoS One 6:e17549. doi: 10.1371/journal.pone.0017549, PMID: 21390233 PMC3048399

[ref13] Cycoń M. Mrozik A. Piotrowska-Seget Z. (2019). Antibiotics in the soil environment—degradation and their impact on microbial activity and diversity. Front. Microbiol. 10:338. doi: 10.3389/fmicb.2019.00338, PMID: 30906284 PMC6418018

[ref14] Delgado-Baquerizo M. Hang-Wei H. Maestre F. T. Guerra C. A. Eisenhauer N. Eldridge D. J.. (2022). The global distribution and environmental drivers of the soil antibiotic Resistome. Microbiome 10:219. doi: 10.1186/s40168-022-01405-w, PMID: 36503688 PMC9743735

[ref15] den Meersche V. Tina G. R. Haesebrouck F. Van Coillie E. Herman L. Van Weyenberg S.. (2019). Presence and fate of antibiotic residues, antibiotic resistance genes and zoonotic Bacteria during biological swine manure treatment. Ecotoxicol. Environ. Saf. 175, 29–38. doi: 10.1016/j.ecoenv.2019.01.127, PMID: 30878661

[ref16] Du L. Liu W. (2012). Occurrence, fate, and Ecotoxicity of antibiotics in agro-ecosystems. A review. Agron. Sustain. Dev. 32, 309–327. doi: 10.1007/s13593-011-0062-9

[ref17] Forsberg K. J. Patel S. Gibson M. K. Lauber C. L. Knight R. Fierer N.. (2014). Bacterial phylogeny structures soil Resistomes across habitats. Nature 509, 612–616. doi: 10.1038/nature13377, PMID: 24847883 PMC4079543

[ref18] Forsberg K. J. Reyes A. Wang B. Selleck E. M. Sommer M. O. A. Dantas G. (2012). The shared antibiotic Resistome of soil Bacteria and human pathogens. Science 337, 1107–1111. doi: 10.1126/science.1220761, PMID: 22936781 PMC4070369

[ref19] Fouz N. Pangesti K. N. A. Yasir M. Al-Malki A. L. Azhar E. I. Hill-Cawthorne G. A.. (2020). The contribution of wastewater to the transmission of antimicrobial resistance in the environment: implications of mass gathering settings. Infect. Dis. Trop. Med. 5:33. doi: 10.3390/tropicalmed5010033, PMID: 32106595 PMC7157536

[ref20] Fresno M. Barrera V. Gornall V. Lillo P. Paredes N. Abalos P.. (2013). Identification of diverse *Salmonella* serotypes, Virulotypes, and antimicrobial resistance phenotypes in waterfowl from Chile. Vector Borne Zoonotic Dis. 13, 884–887. doi: 10.1089/vbz.2013.1408, PMID: 24107205 PMC3868272

[ref21] Fricke W. F. Wright M. S. Lindell A. H. Harkins D. M. Baker-Austin C. Ravel J.. (2008). Insights into the environmental resistance Gene Pool from the genome sequence of the multidrug-resistant environmental isolate *Escherichia Coli* SMS-3-5. J. Bacteriol. 190, 6779–6794. doi: 10.1128/jb.00661-08, PMID: 18708504 PMC2566207

[ref22] Ganesan S. (2022). “Biotic farming using organic fertilizer for sustainable agriculture” in Physical sciences reviews. ed. Zondervan E. (Berlin: Walter de Gruyter)

[ref23] Gillings M. R. (2014). Integrons: past, present, and future. Microbiol. Mol. Biol. Rev. 78, 257–277. doi: 10.1128/MMBR.00056-13, PMID: 24847022 PMC4054258

[ref24] Graham D. W. Bergeron G. Bourassa M. W. Dickson J. Gomes F. Howe A.. (2019). Complexities in understanding antimicrobial resistance across domesticated animal, human, and environmental systems. Ann. N. Y. Acad. Sci. 1441, 17–30. doi: 10.1111/nyas.14036, PMID: 30924539 PMC6850694

[ref25] He L.-Y. He L.-K. Gao F.-Z. Dai-Ling W. Zou H.-Y. Bai H.. (2021). Dissipation of antibiotic resistance genes in manure-amended agricultural soil. Sci. Total Environ. 787:147582. doi: 10.1016/j.scitotenv.2021.147582, PMID: 33992936

[ref26] He T. Wei R.-C. Zhang L. Gong L. Zhu L. Jili G.. (2021). Dissemination of the *Tet* (X)-variant genes from layer farms to manure-receiving soil and corresponding lettuce. Environ. Sci. Technol. 55, 1604–1614. doi: 10.1021/acs.est.0c05042, PMID: 33427447

[ref27] He J. Yan Z. Chen Q. (2020). Transmission of antibiotic resistance genes in agroecosystems: an overview. Front. Agric. Sci. Eng. 7, 329–332. doi: 10.15302/J-FASE-2020333

[ref28] Hendriksen R. S. Bortolaia V. Tate H. Tyson G. H. Aarestrup F. M. McDermott P. F. (2019). Using genomics to track global antimicrobial resistance. Front. Public Health 7:242. doi: 10.3389/fpubh.2019.00242, PMID: 31552211 PMC6737581

[ref29] Heuer H. Solehati Q. Zimmerling U. Kleineidam K. Schloter M. Müller T.. (2011). Accumulation of sulfonamide resistance genes in arable soils due to repeated application of manure containing sulfadiazine. Appl. Environ. Microbiol. 77, 2527–2530. doi: 10.1128/AEM.02577-10, PMID: 21296942 PMC3067416

[ref30] Holvoet K. Sampers I. Callens B. Dewulf J. Uyttendaele M. (2013). Moderate prevalence of antimicrobial resistance in *Escherichia Coli* isolates from lettuce, irrigation water, and soil. Appl. Environ. Microbiol. 79, 6677–6683. doi: 10.1128/AEM.01995-13, PMID: 23974140 PMC3811515

[ref31] Huang J. Mi J. Yan Q. Wen X. Zhou S. Wang Y.. (2021). Animal manures application increases the abundances of antibiotic resistance genes in soil-lettuce system associated with shared bacterial distributions. Sci. Total Environ. 787:147667. doi: 10.1016/j.scitotenv.2021.147667, PMID: 34004530

[ref32] Huong L. Q. Hang T. T. T. Ngoc P. T. Van Tuat C. Erickson V. I. Padungtod P. (2020). Pilot monitoring of antimicrobial residues in chicken and Porkin Vietnam. J. Food Prot. 83, 1701–1706. doi: 10.4315/JFP-20-111, PMID: 32971539

[ref9003] Infante A. San Martín K. (2016). “Manual de producción agroecológica”. Centro de Educación y Tecnología.

[ref33] Iwu C. D. Korsten L. Okoh A. I. (2020). The incidence of antibiotic resistance within and beyond the agricultural ecosystem: a concern for public health. MicrobiologyOpen 9:e1035. doi: 10.1002/mbo3.1035, PMID: 32710495 PMC7520999

[ref34] Jadeja N. B. Worrich A. (2022). From gut to mud: dissemination of antimicrobial resistance between animal and agricultural niches. Environ. Microbiol. 24, 3290–3306. doi: 10.1111/1462-2920.15927, PMID: 35172395

[ref9004] Kalus K. Koziel J. A. Opaliński S. (2019). “A review of biochar properties and their utilization in crop agriculture and livestock production”. Applied Sciences, 9:3494. doi: 10.3390/app9173494

[ref35] Li D. Tao Y. Zhang Y. Yang M. Li Z. Liu M.. (2010). Antibiotic resistance characteristics of environmental Bacteria from an Oxytetracycline production wastewater treatment plant and the Receiving River. Appl. Environ. Microbiol. 76, 3444–3451. doi: 10.1128/AEM.02964-09, PMID: 20400569 PMC2876458

[ref36] Lima T. Domingues S. Da Silva G. J. (2020). Manure as a potential hotspot for antibiotic resistance dissemination by horizontal gene transfer events. Vet. Sci. 7:110. doi: 10.3390/vetsci7030110, PMID: 32823495 PMC7558842

[ref37] Liu B. Kaifeng Y. Ahmed I. Gin K. Xi B. Wei Z.. (2021). Key factors driving the fate of antibiotic resistance genes and controlling strategies during aerobic composting of animal manure: a review. Sci. Total Environ. 791:148372. doi: 10.1016/j.scitotenv.2021.148372, PMID: 34139488

[ref38] Liu W. Ling N. Guo J. Ruan Y. Wang M. Shen Q.. (2021). Dynamics of the antibiotic Resistome in agricultural soils amended with different sources of animal manures over three consecutive years. J. Hazard. Mater. 401:123399. doi: 10.1016/j.jhazmat.2020.123399, PMID: 32763695

[ref39] Makowska N. Koczura R. Mokracka J. (2016). Class 1 integrase, sulfonamide and tetracycline resistance genes in wastewater treatment plant and surface water. Chemosphere 144, 1665–1673. doi: 10.1016/j.chemosphere.2015.10.044, PMID: 26519797

[ref9005] Mshana S. E. Sindato C. Matee M. I. Mboera L. E. (2021). “Antimicrobial use and resistance in agriculture and food production systems in Africa: a systematic review”. Antibiotics, 10:976. doi: 10.3390/antibiotics10080976 34439026 PMC8389036

[ref40] Monteiro M. A. Spisso B. F. Santos J. R. M. P. D. Da Costa R. P. Ferreira R. G. Pereira M. U.. (2016). Occurrence of antimicrobials in river water samples from rural region of the state of Rio de Janeiro, Brazil. J. Environ. Prot. 7, 230–241. doi: 10.4236/jep.2016.72020

[ref41] Pfaffl M. W. (2001). A new mathematical model for relative quantification in real-time RT-PCR. Nucleic Acids Res. 29, 45e–445e. doi: 10.1093/nar/29.9.e45, PMID: 11328886 PMC55695

[ref42] Pu C. Yao Y. Diao J. Gong X. Li J. Sun Y. (2019). Exploring the persistence and spreading of antibiotic resistance from manure to biocompost, soils and vegetables. Sci. Total Environ. 688, 262–269. doi: 10.1016/j.scitotenv.2019.06.081, PMID: 31229823

[ref43] Puvača N. (2022). Antimicrobial resistance and treatment in companion, food and exotic animals. Antibiotics 11:1360. doi: 10.3390/antibiotics11101360, PMID: 36290017 PMC9598238

[ref44] Rani P. Leela K. Balaswamy A. Rao R. Masthan S. C. (2015). Evaluation of integrated nutrient management practices on growth, yield and economics of Green Chilli cv Pusa Jwala (*Capsicum Annuum* L.). Int. J. Bio-Resour. Stress Manag. 6:76. doi: 10.5958/0976-4038.2015.00007.X

[ref45] Reichert G. Hilgert S. Fuchs S. Azevedo J. C. R. (2019). Emerging contaminants and antibiotic resistance in the different environmental matrices of Latin America. Environ. Pollut. 255:113140. doi: 10.1016/j.envpol.2019.113140, PMID: 31541833

[ref46] Retamal P. Fresno M. Dougnac C. Gutierrez S. Gornall V. Vidal R.. (2015). Genetic and phenotypic evidence of the *Salmonella Enterica* serotype Enteritidis human-animal Interface in Chile. Front. Microbiol. 6:464. doi: 10.3389/fmicb.2015.00464, PMID: 26029196 PMC4432690

[ref47] Roca I. Akova M. Baquero F. Carlet J. Cavaleri M. Coenen S.. (2015). The global threat of antimicrobial resistance: science for intervention. New Microbes New Infect. 6, 22–29. doi: 10.1016/j.nmni.2015.02.007, PMID: 26029375 PMC4446399

[ref48] Roe M. Pillai S. (2003). Monitoring and identifying antibiotic resistance mechanisms in Bacteria. Poult. Sci. 82, 622–626. doi: 10.1093/ps/82.4.622 12710483

[ref49] Ruijter J. M. Ramakers C. Hoogaars W. M. H. Karlen Y. Bakker O. Van Den Hoff M. J. B.. (2009). Amplification efficiency: linking baseline and Bias in the analysis of quantitative PCR data. Nucleic Acids Res. 37:e45. doi: 10.1093/nar/gkp045, PMID: 19237396 PMC2665230

[ref50] Shakoor A. Shahzad S. M. Chatterjee N. Arif M. S. Farooq T. H. Altaf M. M.. (2021). Nitrous oxide emission from agricultural soils: application of animal manure or biochar? A global Meta-analysis. J. Environ. Manag. 285:112170. doi: 10.1016/j.jenvman.2021.112170, PMID: 33607561

[ref51] Shawver S. Wepking C. Ishii S. Strickland M. S. Badgley B. D. (2021). Application of manure from cattle administered antibiotics has sustained multi-year impacts on soil Resistome and microbial community structure. Soil Biol. Biochem. 157:108252. doi: 10.1016/j.soilbio.2021.108252

[ref52] Suzuki M. T. Taylor L. T. Delong E. F. (2000). Quantitative analysis of small-subunit rRNA genes in mixed microbial populations via 5Ј-nuclease assays. Appl. Environ. Microbiol. 66, 4605–4614. doi: 10.1128/AEM.66.11.4605-4614.2000 PMC92356 11055900

[ref53] Thakur S. D. Panda A. K. (2017). Rational use of antimicrobials in animal production: a prerequisite to stem the tide of antimicrobial resistance. Curr. Sci. 113:1846. doi: 10.18520/cs/v113/i10/1846-1857

[ref9006] Thanner S. Drissner D. Walsh F. (2016). “Antimicrobial resistance in agricultura”. MBio, 7, 10–1128. doi: 10.1128/mbio.02227-15 PMC4850276 27094336

[ref9007] Urra J. Alkorta I. Garbisu C. (2019). “Potential benefits and risks for soil health derived from the use of organic amendments in agricultura”. Agronomy, 9:542. doi: 10.3390/agronomy9090542

[ref9008] Ventola C. L. (2015). The antibiotic resistance crisis: part 1: causes and threats. Pharmacy and therapeutics, 40:277. 25859123 PMC4378521

[ref54] Vrancianu C. O. Popa L. I. Bleotu C. Chifiriuc M. C. (2020). Targeting plasmids to limit acquisition and transmission of antimicrobial resistance. Front. Microbiol. 11:761. doi: 10.3389/fmicb.2020.00761, PMID: 32435238 PMC7219019

[ref55] Wang N. Hang X. Zhang M. Liu X. Yang H. (2017). Analysis of newly detected tetracycline resistance genes and their flanking sequences in human intestinal Bifidobacteria. Sci. Rep. 7:6267. doi: 10.1038/s41598-017-06595-0, PMID: 28740169 PMC5524971

[ref56] White A. Hughes J. M. (2019). Critical importance of a one health approach to antimicrobial resistance. Eco Health 16, 404–409. doi: 10.1007/s10393-019-01415-5 31250160

[ref57] Xie W.-Y. Shen Q. Zhao F. J. (2018). Antibiotics and antibiotic resistance from animal manures to soil: a review. Eur. J. Soil Sci. 69, 181–195. doi: 10.1111/ejss.12494

[ref58] Yang Q. Gao Y. Ke J. Show P. L. Ge Y. Liu Y.. (2021). Antibiotics: an overview on the environmental occurrence, toxicity, degradation, and removal methods. Bioengineered 12, 7376–7416. doi: 10.1080/21655979.2021.1974657, PMID: 34612807 PMC8806427

[ref59] Zhang M. He L.-Y. Liu Y.-S. Zhao J.-L. Zhang J.-N. Chen J.. (2020). Variation of antibiotic Resistome during commercial livestock manure composting. Environ. Int. 136:105458. doi: 10.1016/j.envint.2020.105458, PMID: 31926439

[ref60] Zhu Y.-G. Johnson T. A. Jian-Qiang S. Qiao M. Guo G.-X. Stedtfeld R. D.. (2013). Diverse and abundant antibiotic resistance genes in Chinese swine farms. Proc. Natl. Acad. Sci. 110, 3435–3440. doi: 10.1073/pnas.1222743110, PMID: 23401528 PMC3587239

